# Association of co-administration of neurokinin-1 receptor antagonists and dexamethasone with new-onset diabetes in patients receiving chemotherapy: a retrospective cross-sectional study using health claims database in Japan

**DOI:** 10.1007/s00520-026-11414-2

**Published:** 2026-10-29

**Authors:** Yuki Ozawa, Shinya Suzuki, Takuma Koinuma, Takashi Yokokawa, Takenori Ichimura, Kenichi Suzuki, Yusuke Hori, Shinobu Imai

**Affiliations:** 1 Department of Clinical Pharmacology, School of Pharmacy, Tokyo University of Pharmacy and Life Sciences, Tokyo, Japan; 2 Department of Pharmacy, Kanagawa Prefectural Keiyukai, Keiyu Hospital, Kanagawa, Japan; 3 Drug Informatics Group, Laboratory of Clinical Pharmacy, School of Pharmacy, Tokyo University of Pharmacy and Life Sciences, 1432-1, Horinouchi, Hachioji-City, Tokyo, 192-0392 Japan; 4 Division of Pharmacoepidemiology, Showa Medical University Graduate School of Pharmacy, Tokyo, Japan

**Keywords:** Neurokinin-1 receptor antagonist, Dexamethasone, Diabetes, Drug-drug interaction, Chemotherapy

## Abstract

**Purpose:**

We aimed to optimize antiemetic strategies by evaluating the association of co-administration of neurokinin-1 receptor antagonist (NK1-RA) administration and dexamethasone on new-onset diabetes in patients receiving chemotherapy.

**Methods:**

This retrospective cross-sectional study was conducted using the DeSC database and included patients aged ≥ 18 years with solid tumors who initiated parenteral anticancer agents and dexamethasone between April 2014 and March 2023. Patients with a history of diabetes or hematologic malignancies were excluded. The primary outcome was new-onset diabetes during the 2 months following the index month, defined by International Classification of Diseases, Tenth Revision codes or prescription data. Multivariable logistic regression assessed the association between co-administration of NK1-RA and dexamethasone and new-onset diabetes.

**Results:**

Among 71,539 eligible patients, 51.1% received NK1-RAs. Co-administration of NK1-RA and dexamethasone was significantly associated with an increased risk of new-onset diabetes (adjusted odds ratio: 1.43; 95% confidence interval: 1.31–1.55; *p* < 0.001). Sensitivity analyses demonstrated consistent results across different evaluation periods and outcome definitions.

**Conclusion:**

Co-administration of NK1-RA and dexamethasone was associated with an increased risk of new-onset diabetes. These findings highlight a previously unrecognized safety signal and support the careful management of dexamethasone, as recommended by many clinical guidelines.

**Supplementary Information:**

The online version contains supplementary material available at https://doi.org/10.1007/s00520-026-11414-2.

## Introduction

Chemotherapy-induced nausea and vomiting (CINV) is a major adverse event of chemotherapy and reduces patients’ quality of life [1]. Recent advances in antiemetic therapy have improved CINV control. The overall complete response rate for cisplatin-based highly emetogenic chemotherapy (HEC) reached 78% with the use of four antiemetic agents—olanzapine, neurokinin-1 receptor antagonist (NK1-RA), 5-hydroxytryptamine type 3 serotonin receptor antagonist (5HT3-RA), and dexamethasone—compared with 52.3% 20 years ago [2, 3]. With improved CINV control, attention has shifted to the risks associated with antiemetics, promoting strategies such as steroid sparing [4–6]. Dexamethasone, when used as an antiemetic during chemotherapy, causes several adverse effects, including diabetes, decreased bone mineral density, insomnia, and agitation [7–9]. In addition to steroid-related adverse effects, drug–drug interactions (DDIs) involving NK1-RAs are another major concern.

NK1-RAs are moderate cytochrome P450 3A4 (CYP3A4) inhibitors, and clinically significant DDIs have been reported, including respiratory depression in patients receiving oxycodone with an NK1-RA [10] and severe somnolence with quetiapine [11]. These events are likely attributable to increased drug concentrations resulting from CYP3A4 inhibition. In addition, a systematic review of DDIs related to aprepitant and fosaprepitant indicates that dexamethasone, which is routinely co-administered with an NK1-RA, is a particularly significant interaction pair [12]. This combination doubled the area under the concentration–time curve of dexamethasone [13]. Many international guidelines, such as those from the American Society of Clinical Oncology and the National Comprehensive Cancer Network, recommend adjusted dexamethasone doses for HEC regimens [14, 15]. However, recommendations regarding dexamethasone dose reduction are not entirely consistent across guidelines. For example, the Multinational Association of Supportive Care in Cancer and European Society for Medical Oncology Joint Guideline does not provide specific recommendations on dexamethasone dose reduction [16]. Moreover, for less than moderately emetogenic chemotherapy, there is even less consensus regarding optimal antiemetic strategies. This variation in recommendations may partly reflect the limited clinical evidence available beyond pharmacokinetic studies [13]; therefore, the clinical consequences of the DDI between NK1-RAs and dexamethasone remain unclear. New safety signal of these clinical outcomes would provide a rationale for optimizing antiemetic strategies.

A representative adverse effect of dexamethasone is diabetes. A previous study suggests that even short-term administration as an antiemetic can cause diabetes, and the risk increases with higher cumulative doses [7]. Therefore, we hypothesized that increased dexamethasone concentrations resulting from NK1-RA co-administration might impact the development of diabetes. A previous meta-analysis also suggests an association between diabetes and cancer mortality [17], underscoring its clinical significance.

We aimed to optimize antiemetic strategies by evaluating the association of co-administration of NK1-RA and dexamethasone for CINV with new-onset diabetes using large-scale health claims data.

## Methods

### Study design and setting

**Fig. 1:**
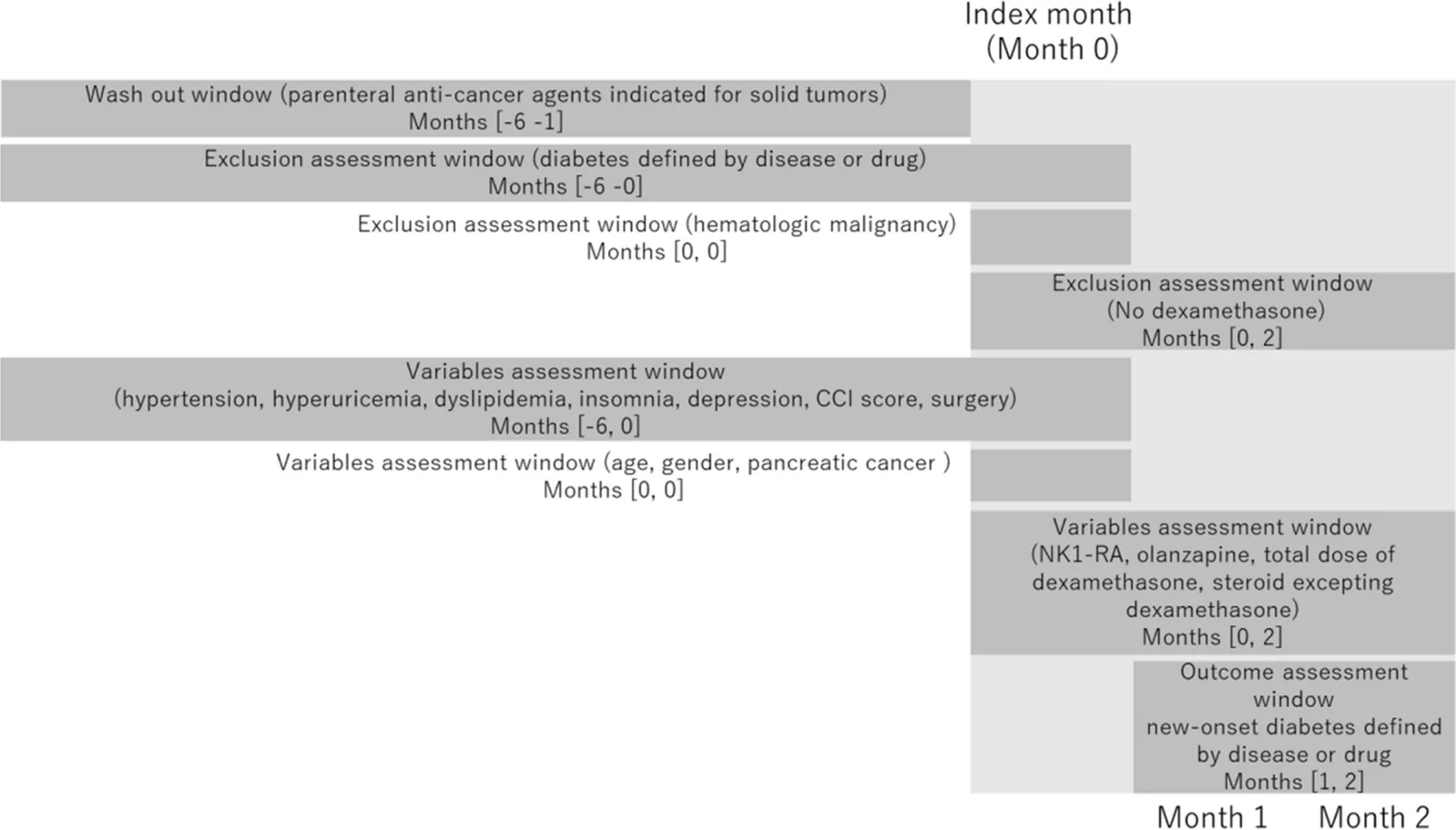
Design diagram. *CCI* Charlson comorbidity index, *NK1-RA* neurokinin 1 receptor antagonist

This retrospective cross-sectional study was conducted using the DeSC database (DeSC Healthcare Inc., Japan). Several studies have used this database [18–20]. The database contains anonymized claims data for over 3 million patients with cancer recorded between April 2014 and October 2023. It includes three insurance systems: health insurance for corporate employees, National Health Insurance for self-employed and unemployed individuals, and the Medical Care System for the Advanced Elderly for those aged ≥ 75 years. Individual patients are uniquely identified across insurers through health care institutions, allowing longitudinal tracking. The patient enrollment period spanned from April 2014 to March 2023, with the outcome assessment period defined as the 2 months following the index month. The study design diagram is shown in Fig. 1. Because we evaluated the association within a fixed short-term window without considering censoring, it was defined as cross-sectional, although new-onset diabetes was assessed after chemotherapy initiation. The study protocol was approved by the Ethics Committee of Tokyo University of Pharmacy and Life Sciences (approval number: 2025–033). This study followed the Reporting of Studies Conducted Using Observational Routinely Collected Health Data for Pharmacoepidemiology (RECORD-PE) statement.

### Participants

Eligibility criteria included patients aged ≥ 18 years with solid tumors who received parenteral anticancer agents along with dexamethasone. These anticancer agents were identified using Anatomical Therapeutic Chemical (ATC) code L and were individually reviewed to ensure clinical relevance. The index month was defined as the first month in which a patient received these anticancer agents within the database between April 2014 and March 2023. We adopted a new-user design with a 6-month washout period to eliminate prevalent user bias. Exclusion criteria were: (1) diabetes during the index month or in the 6 months prior, (2) hematologic malignancy during the index month, and (3) no dexamethasone administration during the 3 months after the index month. Diabetes was defined using International Classification of Diseases 10th Revision (ICD-10) codes (E10.x–E14.x) or prescription data (ATC code A10). Hematologic malignancy was defined using ICD-10 codes for malignant neoplasms (C81.x–C96.x).

### Outcomes

The primary outcome was new-onset diabetes during the 2 months following the index month. Based on a previous validation study [21], new-onset diabetes was defined using ICD-10 codes (E11.x–E14.x) excluding type 1 diabetes, or prescription data (ATC code A10). To exclude prevalent cases, patients with diabetes diagnosis during the index month were excluded. Accordingly, incidence was evaluated over the subsequent two months (month 1 and month 2). The assessment period of 3 months was defined based on a previous study reporting steroid-induced diabetes after antiemetic dexamethasone therapy in chemotherapy-treated cancer patients [7]. Secondary outcomes included the frequency and timing of new-onset diabetes.

### Variables

Variables that could potentially affect the outcome were selected. Age was categorized as < 70 or ≥ 70 years at the index month. Sex was classified as male or female. Comorbidities, including hypertension, hyperuricemia, dyslipidemia, depression, and insomnia, were defined using ICD-10 codes during the index month or in the 6 months prior (Online Resource 1). Pancreatic cancer was identified using ICD-10 codes (C25.x) during the index month to define the population initiating chemotherapy. The Charlson Comorbidity Index (CCI) was calculated based on a previously validated study [22], using ICD-10 codes recorded during the index month or in the 6 months prior (Online Resource 2). In addition, because all participants in this study were patients with cancer, "Cancer" and "Metastatic carcinoma" were excluded from the CCI score calculation to improve the interpretability of the results. NK1-RAs, including aprepitant, fosaprepitant, and fosnetupitant, were identified using ATC code A04AD. Concomitant medications included olanzapine and systemic steroids other than dexamethasone, identified using ATC codes (N05AH03; H02AB excluding H02AB02 [dexamethasone] and H02AB08 [triamcinolone]) during the index month and the subsequent 2 months. Dexamethasone was identified using ATC code H02AB02, and the total dose during the index month and subsequent 2 months was calculated. Surgery was defined using treatment codes for general anesthesia and endotracheal intubation recorded during the index month or in the 6 months prior.

Most variables were selected based on previous studies [23–27], while others were chosen based on clinical relevance. Specifically, the CCI score was included as a validated measure of overall comorbidity burden. Olanzapine was included as an exploratory variable because its use is contraindicated in patients with diabetes in Japan and may influence both prescribing patterns and diabetes risk.

### Statistical analysis

Continuous variables were summarized using the mean and standard deviation. Categorical variables were summarized as frequencies and percentages. Missing data were not imputed because the study relied on claims-based diagnostic and procedural codes, for which the absence of a code was assumed to indicate the absence of the condition or treatment. For the primary analysis, multivariable logistic regression was used to assess the association between co-administration of NK1-RA and dexamethasone and new-onset diabetes. Odds ratios (ORs), 95% confidence intervals (CIs), and p-values were calculated for each variable. New-onset diabetes was defined as the dependent variable, and the covariates described above were included as independent variables. Subgroup analyses were performed by dividing the population into cisplatin and non-cisplatin groups, as well as by stratifying patients based on the specific type of NK1-RA administered (aprepitant, fosaprepitant, and fosnetupitant). Sensitivity analyses were conducted by (1) restricting the outcome definition to both ICD-10 codes and prescription data to increase diagnostic specificity, (2) extending the assessment period to 6 months, (3) excluding covariates with high ORs, and (4) broadening the classification of olanzapine to a wider group of second-generation antipsychotics (quetiapine, clozapine, and risperidone) known for increasing the risk of diabetes [28]. Due to the exploratory nature of this study, a sample size calculation was not performed. A two-sided *p*-value < 0.05 was considered statistically significant. All statistical analyses were performed using R software, version 4.5.0.

## Results

### Participants flow and characteristics

**Fig. 2:**
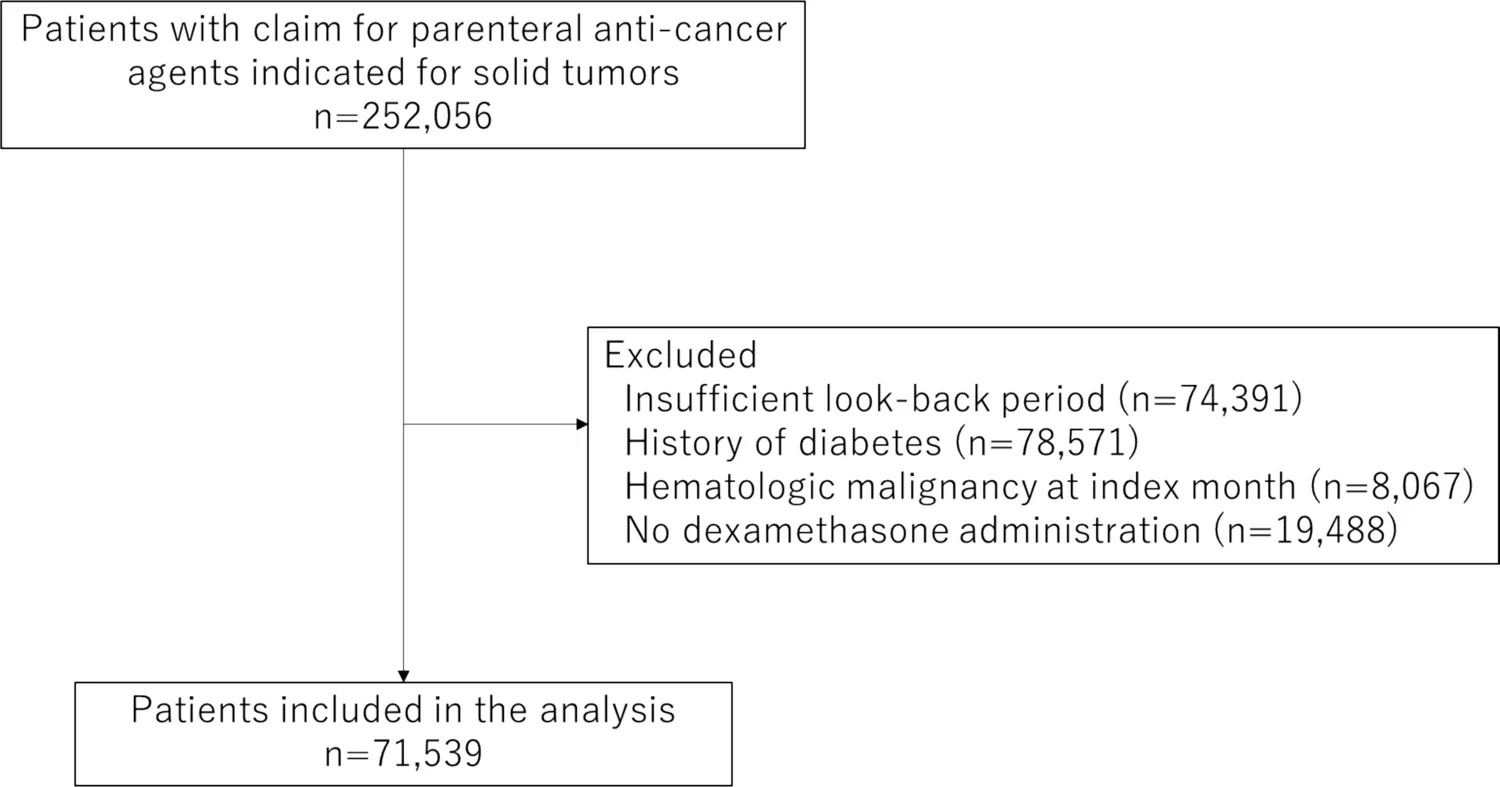
Participant flow diagram

The participant flow is shown in Fig. 2. During the enrollment period, 252,056 patients were initially identified. Of these, 180,517 patients were excluded: 74,391 were excluded due to an insufficient look-back period; 78,571 had a history of diabetes; 8,067 had hematologic malignancies; and 19,488 had not received dexamethasone. Consequently, 71,539 patients were included in the analysis. Patient characteristics are summarized in Table 1. During the 3-month assessment period, the mean duration of chemotherapy was 2.51 months. NK1-RAs were administered to 51.1% of patients, with a mean duration of 2.32 months.

**Table 1:** Participant characteristics

	*N* = 71,539
Age (years), mean (SD)	69.6 (10.6)
Sex (male/female), n (%)	36,332 (50.8)/35,207 (49.2)
Comorbidity	
Hypertension, n (%)	33,735 (47.2)
Hyperuricemia, n (%)	6,443 (9.0)
Dyslipidemia, n (%)	22,173 (31.0)
Depression, n (%)	3,731 (5.2)
Insomnia, n (%)	19,193 (26.8)
Pancreatic cancer, n (%)	4,796 (6.7)
CCI score without cancer ≥ 1, n (%)	46,967 (65.7)
Concomitant medication	
NK1-RA, n (%)	36,583 (51.1)
Olanzapine, n (%)	4,841 (6.8)
Total dose of dexamethasone, mg mean (SD)	51.2 (35.8)
Steroid except dexamethasone, n (%)	9,928 (13.9)
Surgery before chemotherapy, n (%)	25,489 (35.6)

*CCI* Charlson comorbidity index, *NK1-RA* neurokinin 1 receptor antagonist, *SD* standard deviation

### Primary outcome

The association between co-administration of NK1-RA and dexamethasone and new-onset diabetes is shown in Table 2. NK1-RA administration was significantly associated with new-onset diabetes (OR: 1.43; 95% CI: 1.31–1.55).

**Table 2:** Multivariable logistic regression analysis for new-onset diabetes

	Odds ratio	95% confidence interval	*p*-value
Age ≥ 70	1.07	0.98–1.16	0.142
Sex (female)	0.69	0.63–0.75	< 0.001
Hypertension	1.23	1.13–1.34	< 0.001
Hyperuricemia	1.03	0.90–1.16	0.682
Dyslipidemia	1.11	1.02–1.22	0.015
Depression	1.01	0.84–1.20	0.93
Insomnia	0.92	0.84–1.00	0.061
Pancreatic cancer	4.19	3.77–4.66	< 0.001
CCI score without cancer ≥ 1	1.15	1.05–1.26	0.003
NK1-RA	1.43	1.31–1.55	< 0.001
Olanzapine	1.15	0.99–1.33	0.06
Total dose of dexamethasone ≥ 50 mg	1.09	1.00–1.19	0.04
Steroid except dexamethasone	3.04	2.79–3.30	< 0.001
Surgery before chemotherapy	0.51	0.46–0.57	< 0.001

*CCI* Charlson comorbidity index, *NK1-RA* neurokinin 1 receptor antagonist

Other factors significantly associated with new-onset diabetes included hypertension, dyslipidemia, pancreatic cancer, a CCI score excluding cancer ≥ 1, a total dexamethasone dose ≥ 50 mg, and the use of systemic steroids other than dexamethasone. In contrast, female sex and prior surgery were significantly associated with a decreased risk of new-onset diabetes.

### Secondary outcomes

Regarding the frequency and timing of new-onset diabetes, 2,826 patients (4.0%) developed diabetes during the assessment period. Of these, 1,366 cases occurred in the month following the index month (month 1), and 1,460 cases occurred in the subsequent month (month 2).

### Subgroup analysis

Among the 71,539 patients included in the analysis, 15,141 patients (21.1%) received at least one dose of cisplatin during the 3-month observation period. Moreover, NK1-RAs were administered to 13,584 patients (89.7%) of the cisplatin group and 22,999 patients (40.8%) of the non-cisplatin group. The incidence of new-onset diabetes was 5.59% (846 patients) in the cisplatin group and 3.51% (1,980 patients) in the non-cisplatin group. In both the cisplatin group and the non-cisplatin group, NK1-RA administration was significantly associated with new-onset diabetes (OR: 1.95; 95% CI: 1.44–2.65 and OR: 1.12; 95% CI: 1.01–1.24, respectively; Online Resource 3 and 4).

Moreover, in a subgroup analysis according to the specific NK1-RA administered, first, 766 patients who received more than one type of NK1-RA during the 3-month observation period were excluded. The numbers of patients and new-onset diabetes cases for each agent were 27,819 (963 patients, 3.46%) for aprepitant, 7,530 (445 patients, 5.91%) for fosaprepitant, and 602 (24 patients, 3.99%) for fosnetupitant. NK1-RA administration was significantly associated with the development of diabetes in the aprepitant group (OR: 1.32; 95% CI: 1.20–1.45; Online Resource 5) and the fosaprepitant group (OR: 1.91; 95% CI: 1.69–2.16; Online Resource 6). In the fosnetupitant group, the association was not statistically significant (OR: 1.47; 95% CI: 0.97–2.24; Online Resource 7).

### Sensitivity analysis

Five sensitivity analyses were conducted. When the definition of new-onset diabetes was narrowed to cases identified by both ICD-10 codes and prescription data, NK1-RA administration remained significantly associated with new-onset diabetes (OR: 1.36; 95% CI: 1.13–1.64; Online Resource 8). Similarly, significant associations were observed when the assessment period was extended to 6 months (OR: 1.37; 95% CI: 1.29–1.46; Online Resource 9), when covariates with high ORs, including pancreatic cancer or systemic steroids other than dexamethasone, were excluded (OR: 1.14; 95% CI: 1.05–1.23, and OR: 1.39; 95% CI: 1.28–1.51, respectively; Online Resources 10 and 11), and when broadening the classification of olanzapine to a wider group of second-generation antipsychotics (OR: 1.40; 95% CI: 1.29–1.53; Online Resources 12).

Across all sensitivity analyses, pancreatic cancer, systemic steroids other than dexamethasone, and the CCI score remained consistently associated with new-onset diabetes, while female sex and prior surgery remained significantly associated with a decreased risk (Online Resources 8–12).

## Discussion

Our findings suggest that co-administration of NK1-RA and dexamethasone for CINV is associated with an increased risk of new-onset diabetes, and this demonstrated high robustness across multiple sensitivity analyses. To our knowledge, this study is the first to investigate the association between co-administration of NK1-RA and dexamethasone for CINV and new-onset diabetes. These findings highlight a previously unrecognized safety signal associated with NK1-RAs and the importance of the careful management of dexamethasone.

The cross-sectional design limits causal inference; however, several considerations support the plausibility of the observed association. Previous studies have identified several risk factors for CINV; however, diabetes has not been reported as one of them [29]. Furthermore, current clinical guidelines do not recommend prioritizing NK1-RA administration or intensifying antiemetic therapy specifically for patients with diabetes [14–16]. Therefore, baseline selection bias is unlikely. In addition, among patients receiving NK1-RAs, the duration of administration accounted for most of the 3-month assessment period, further supporting the observed association between NK1-RA administration and new-onset diabetes.

The frequency of diabetes during HEC (57.1%) and moderately emetogenic chemotherapy (42.9%) has been reported in a previous study [7]. In that study, 14.3% of patients with cancer and no history of diabetes developed diabetes within 3 months after starting chemotherapy. Furthermore, 50.6% of patients developed insulin resistance, compared with 28.6% at baseline. These rates are higher than those observed in our study (4.0%), likely because of differences in data collection methods. We relied on health claims data, whereas the previous study used blood test data, such as homeostasis model assessment of insulin resistance, fasting plasma glucose, 2-h postprandial glucose, and hemoglobin A1c. Therefore, if laboratory data had been available in our analysis, additional cases of new-onset diabetes might have been identified, potentially resulting in a higher OR. However, this possibility remains unclear.

Our proposed mechanism is that the DDI between NK1-RAs and dexamethasone increases dexamethasone concentrations. Co-administration of NK1-RAs has been reported to double the area under the concentration–time curve of dexamethasone through inhibition of CYP3A4 [13]. As noted earlier, dexamethasone used as an antiemetic drug during chemotherapy can lead to the development of diabetes [7]. Therefore, increased dexamethasone exposure may facilitate the development of diabetes as an adverse effect. This pharmacokinetic interaction may have contributed to the association observed in our study. In basic research, the NK1 receptor and its ligand, substance P, are involved in blood glucose regulation [30]. However, the underlying mechanisms are complex and remain controversial. Some studies suggest that NK1-RAs may prevent diabetes [31], while others report a potential increase in risk [32]. Therefore, the direct effect of NK1-RAs on glucose metabolism remains unclear. Taken together, altered dexamethasone exposure related to the known pharmacokinetic interaction between NK1-RAs and dexamethasone may be one possible but unproven explanation for the observed association with new-onset diabetes. However, although cumulative dexamethasone dose was included as a covariate in our analyses, detailed dosing and administration schedules, and actual dexamethasone exposure could not be fully assessed. In addition, differences in glucocorticoid exposure, chemotherapy and antiemetic treatment intensity, and other residual confounding may also have contributed to the observed association. Therefore, whether altered dexamethasone exposure associated with NK1-RA co-administration mediates the development of new-onset diabetes cannot be determined from the present study and requires further investigation.

In the subgroup analysis stratified by cisplatin exposure, the co-administration of NK1-RAs and dexamethasone was associated with an increased risk of new-onset diabetes in both the cisplatin and non-cisplatin groups. However, the magnitude of the association differed between the cisplatin and non-cisplatin subgroups. In addition, all individual types of NK1-RAs showed a similar trend toward an increased risk, although fosnetupitant did not reach statistical significance. As fosnetupitant was launched only recently (in 2022) in Japan, it was administered to only 602 patients in the current study. This smaller sample size may have limited the statistical power to detect an association. Therefore, further evaluation will be required in the future as the prescription volume of fosnetupitant increases.

This study has some limitations. First, because of the nature of health claims data, we could not adjust for important variables such as body mass index, diet, exercise habits, and family history of diabetes. In addition, this database provides prescription information on a monthly basis, allowing us to obtain information on the dosage and duration of each prescription. However, because the data are aggregated by month and do not include the exact prescription dates, it is not possible to determine the order of prescriptions within the same month or whether medications were administered continuously or intermittently. Consequently, we were unable to capture the concomitant chemotherapy regimens and detailed antiemetic strategies, such as regimen-specific dexamethasone dosing or tapering approaches. In addition, we could not fully track the overall combinations of other medications, such as the co-administration of dexamethasone with CYP3A4 inhibitors other than NK1-RAs. Furthermore, patients with cancer may be more likely to develop insulin resistance through multiple mechanisms, including the effects of tumor-derived inflammatory cytokines [33]. However, cancer-specific characteristics, including disease stage and severity, could not be assessed in the present study. These residual confounding factors are the major limitations of this study. Second, the use of a washout period to define new users may have reduced the number of patients aged ≥ 75 years, when many patients transition to the Medical Care System for the Advanced Elderly. This might have resulted in slight differences between our study population and the general age distribution. Third, as discussed in the Discussion section, because new-onset diabetes was defined using only diagnostic codes and prescription data, the actual incidence of diabetes may be underestimated.

In conclusion, we suggest an association between co-administration of NK1-RA and dexamethasone for CINV and new-onset diabetes. These findings highlight a previously unrecognized safety signal and support the careful management of dexamethasone, as recommended by many clinical guidelines. Given the potential clinical implications for antiemetic decision-making, future research is needed to clarify causality using alternative study designs, such as cohort studies, as well as to identify patient populations at high risk for developing diabetes.

## Supplementary Information

Below is the link to the electronic supplementary material.

Supplementary file1 (DOCX 19.5 KB)

Supplementary file2 (DOCX 21.2 KB)

Supplementary file3 (DOCX 20.0 KB)

Supplementary file4 (DOCX 19.8 KB)

Supplementary file5 (DOCX 22.0 KB)

Supplementary file6 (DOCX 22.2 KB)

Supplementary file7 (DOCX 22.3 KB)

Supplementary file8 (DOCX 21.2 KB)

Supplementary file9 (DOCX 21.2 KB)

Supplementary file10 (DOCX 21.1 KB)

Supplementary file11 (DOCX 21.1 KB)

Supplementary file12 (DOCX 22.6 KB)

## Data Availability

The data that support the findings of this study are available from the corresponding author upon reasonable request.
