# Supplementary material for: Association of co-administration of neurokinin-1 receptor antagonists and dexamethasone with new-onset diabetes in patients receiving chemotherapy: a retrospective cross-sectional study using health claims database in Japan
Source: Support Care Cancer. 2026 Oct 29;34(11):1166. doi: 10.1007/s00520-026-11414-2 (PMC13719788; doi:10.1007/s00520-026-11414-2)
Supplement: Supplementary file 4 [file 520_2026_11414_MOESM1_ESM.docx]

**Article title: Association of Co-administration of Neurokinin-1 Receptor Antagonists and Dexamethasone with New-Onset diabetes in Patients Receiving Chemotherapy: A Retrospective Cross-Sectional Study Using Health Claims Database in Japan**

**Journal name: *Supportive Care in Cancer***

**Authors:**

Yuki Ozawa^1,2^, Shinya Suzuki ^3^*, Takuma Koinuma^3^, Takashi Yokokawa^1^, Takenori Ichimura^3^, Kenichi Suzuki^1^, Yusuke Hori^3^, and Shinobu Imai^4^

**Affiliation**s**:**

1.	Department of Clinical Pharmacology, School of Pharmacy, Tokyo University of Pharmacy and Life Sciences, Tokyo, Japan

2.	Department of Pharmacy, Kanagawa Prefectural Keiyukai, Keiyu Hospital, Kanagawa, Japan

3.	Drug Informatics Group, Laboratory of Clinical Pharmacy, School of Pharmacy, Tokyo University of Pharmacy and Life Sciences, Tokyo, Japan

4.	Division of Pharmacoepidemiology, Department of Healthcare and Regulatory Sciences, Showa University School of Pharmacy, Tokyo, Japan

**Corresponding author:**

Shinya Suzuki, Drug Informatics Group, Laboratory of Clinical Pharmacy, School of Pharmacy, Tokyo University of Pharmacy and Life Sciences, 1432-1, Horinouchi, Hachioji-city, Tokyo 192-0392, Japan

Tel: +08- 42-676-4364

E-mail: ssuzuki@toyaku.ac.jp

**Online Resource 1. ICD-10 codes of comorbidity**

| Comorbidity | ICD-10 codes |
| --- | --- |
| Hypertension | I10–I15.x |
| Hyperuricemia | E79.0 |
| Dyslipidemia | E78.0–78.5, E78.8–9 |
| Depression | F32.x–F33.x |
| Insomnia | G47.0 |
| Pancreatic cancer | C25.x |

*ICD* international classification of diseases.
